# Universal Hepatitis B Vaccination in Adults Aged 19–59 Years: Updated Recommendations of the Advisory Committee on Immunization Practices — United States, 2022

**DOI:** 10.15585/mmwr.mm7113a1

**Published:** 2022-04-01

**Authors:** Mark K. Weng, Mona Doshani, Mohammed A. Khan, Sharon Frey, Kevin Ault, Kelly L. Moore, Eric W. Hall, Rebecca L. Morgan, Doug Campos-Outcalt, Carolyn Wester, Noele P. Nelson

**Affiliations:** ^1^Division of Viral Hepatitis, National Center for HIV, Viral Hepatitis, STD, and TB Prevention, CDC; ^2^Saint Louis University School of Medicine, St. Louis, Missouri; ^3^University of Kansas Medical Center, Kansas City, Kansas; ^4^Immunize.org, Saint Paul, Minnesota; ^5^School of Public Health, Oregon Health & Science University, Portland, Oregon; ^6^Department of Health Research Methods, Evidence, and Impact, McMaster University, Hamilton, Ontario, Canada; ^7^College of Medicine and Public Health, University of Arizona, Phoenix, Arizona.

Hepatitis B (HepB) vaccines have demonstrated safety, immunogenicity, and efficacy during the past 4 decades (*1*,*2*). However, vaccination coverage among adults has been suboptimal, limiting further reduction in hepatitis B virus (HBV) infections in the United States. This Advisory Committee on Immunization Practices (ACIP) recommendation expands the indicated age range for universal HepB vaccination to now include adults aged 19–59 years. Removing the risk factor assessment previously recommended to determine vaccine eligibility in this adult age group (*2*) could increase vaccination coverage and decrease hepatitis B cases. 

## Background

Hepatitis B is a vaccine-preventable, communicable disease of the liver caused by HBV. HBV is transmitted through percutaneous (i.e., puncture through the skin) or mucosal (i.e., direct contact with mucous membranes) exposure to infectious blood or body fluids. Since HepB vaccine was introduced in 1982, the number of reported hepatitis B cases has declined substantially. However, despite reductions in hepatitis B incidence during the past 4 decades, which were achieved through incremental expansion of groups for whom HepB vaccination is recommended, progress in recent years on further reducing acute hepatitis B cases has stalled (*3*). Incident hepatitis B declined from 26,654 reported cases (172,700 estimated actual cases) in 1985 to a low of 2,791 reported cases (18,100 estimated actual cases) in 2014 (*3*,*4*). In 2019, a total of 3,192 cases of acute hepatitis B were reported to CDC, corresponding to 20,700 estimated acute infections (95% CI = 11,800–50,800). The most commonly reported risk behaviors and exposures were injection drug use (35%), multiple sex partners (23%), and surgery (10%), followed by other sexual and bloodborne risk behaviors; risk behavior and exposure information were missing for 37.1% of cases. There are an estimated 880,000 (95% CI = 580,000–1,170,000) prevalent chronic HBV infections in the United States based on 2013–2018 National Health and Nutrition Examination Survey data, with a modeled estimate of 1.89 million (range = 1.49–2.40 million) that accounts for potential underrepresentation of the non-U.S.–born population (*5*,*6*). In 2018, the reported HepB vaccination coverage (≥3 doses) was 30.0% among adults aged ≥19 years, only a small increase over the past 4 decades (*7*).

## Methods

During September 2019–October 2021, the ACIP* Hepatitis Work Group^†^ (Work Group) held monthly conference calls to review and discuss scientific evidence relevant to the use of HepB vaccines in a universal adult vaccination recommendation. The Work Group identified the following outcomes of interest for evaluation: incidence of hepatitis B, morbidity related to hepatitis B, mortality related to hepatitis B, and vaccine-related serious adverse events. Data on universal HepB vaccination outcomes and safety were summarized based on findings from a systematic review of the literature completed on September 10, 2020, and updated September 20, 2021. The Work Group assessed the certainty of evidence at the outcome level related to the U.S.-licensed HepB vaccines for all adults previously unvaccinated against HBV infection, using the Grading of Recommendations Assessment, Development, and Evaluation (GRADE) approach. Detailed descriptions of methods and results are available in the GRADE evidence profile (https://www.cdc.gov/vaccines/acip/recs/grade/hepb-adults.html). After the GRADE assessment, decisions were made using the Evidence to Recommendation (EtR) Framework (https://www.cdc.gov/vaccines/acip/recs/grade/hepb-adults-etr.html).

During July 2021–February 2022, the Work Group participated in three conference calls to review the evidence for the seroprotection and safety of PreHevbrio, a three-antigen 3-dose HepB vaccine newly approved by the Food and Drug Administration (FDA) in 2021. Description of the methods and results are available for the GRADE evidence (https://www.cdc.gov/vaccines/acip/recs/grade/prehevbrio-hepb.html) and EtR Framework (https://www.cdc.gov/vaccines/acip/recs/grade/prehevbrio-hepb-etr.html).

## Summary of Key Findings

The scientific literature was searched through a systematic review using PubMed, Medline, Embase, CINAHL, and Cochrane Library databases from January 1, 2006, through September 10, 2020. Search terms included “hepatitis b vaccines,” “adult,” “routine,” and “universal.” To qualify as a candidate for inclusion in the review, a study had to discuss adult HepB vaccination. Studies were excluded if they did not address the adult population, were non-English language, discussed HepB vaccines not licensed in the United States, or if data could not be abstracted. The search identified 3,226 studies, 263 of which were deemed eligible and informed this review. Rates of reported acute hepatitis B have not notably decreased for over 1 decade, with 20,700 estimated infections in 2019 (*3*,*4*). None of the identified studies reported hepatitis B incidence, morbidity, and mortality when comparing universal and risk-based adult HepB vaccination. The safety of single-antigen 3-dose HepB vaccines has been established (*1*,*2*). PreHevbrio was approved by FDA in 2021 and recommended by ACIP in 2022. Little or no difference in seroprotection or occurrence of serious adverse events or mild adverse events (GRADE evidence type 3; low certainty evidence) was found for PreHevbrio in comparison with a 3-dose, single-antigen vaccine (Engerix-B), and serious adverse events were rare for both vaccines. The 2-dose HepB vaccine (Heplisav-B) was approved by FDA in 2017 and recommended by ACIP in 2018. No difference in occurrence of serious adverse events (GRADE evidence type 1; high certainty evidence) was found for Heplisav-B compared with a 3-dose vaccine (Engerix-B), and serious adverse events were rare for both vaccines (*8*).

## Rationale for Recommendations

Approximately one half of acute hepatitis B cases reported in 2019 occurred among persons aged 30–49 years (Figure). The number of cases of acute hepatitis B has increased among adults aged ≥40 years, particularly among those aged 40–49 years, for whom the rate of reported cases increased from 1.9 per 100,000 population in 2011 to 2.7 per 100,000 population in 2019 (Figure). The rate among adults aged 50–59 years increased 45.5% during the same period (from 1.1 to 1.6 per 100,000 population) and accounted for 22.2% of reported cases in 2019. Acute HBV infections among adults leads to chronic hepatitis B disease in an estimated 2%–6% of cases.

**FIGURE:**
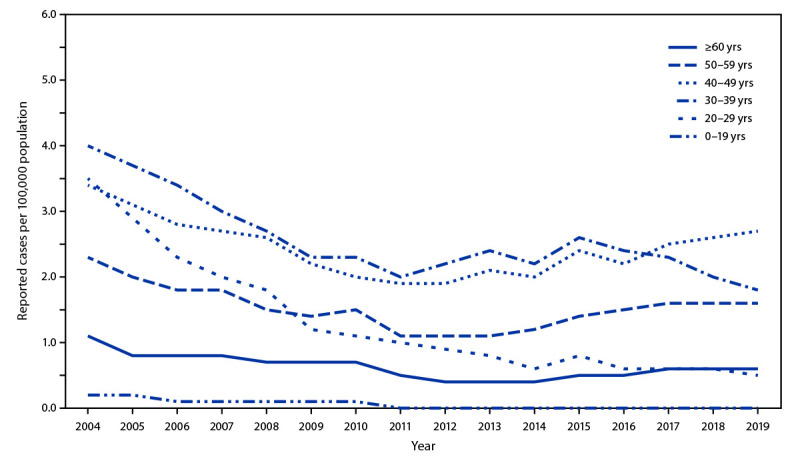
Rates of reported acute hepatitis B virus infection, by age group — United States, 2004–2019 **Source:** https://www.cdc.gov/hepatitis/statistics/2019surveillance/Figure2.4.htm

HepB vaccination coverage among adults aged ≥19 years is low. In 2018, self-reported HepB vaccination coverage (≥3 doses) among adults aged ≥19 years was 30.0% (*7*). HepB vaccination coverage (≥3 doses) was 40.3% for adults aged 19–49 years and 19.1% for adults aged ≥50 years. During 2013–2018, 21.4% (95% CI = 20.2%–22.6%) of adults aged ≥25 years had vaccine-induced immunity to hepatitis B (*5*).

HepB vaccination coverage among adults with risk factors has been suboptimal. In 2018, self-reported coverage (≥3 doses) was 33.0% among adults with chronic liver disease, 38.9% among travelers to countries where HBV infections have been endemic since 1995, 33.0% among adults with diabetes aged 19–59 years, and 67.2% among health care personnel (*7*). In a national survey of 433 family medicine physicians and 420 internal medicine physicians to assess their barriers to adult HepB vaccination, 68% of physicians cited patients’ nondisclosure of risk factors as a barrier, and 44% felt there was inadequate time to routinely assess patients for risk factors (*9*).

A universal recommendation for HepB vaccination could increase the number of persons who receive vaccination before the onset of chronic liver disease and other comorbidities (e.g., obesity or diabetes) that might make vaccination less effective. For example, patients with chronic liver disease are known to have decreased immune response to HepB vaccination (*10*).

Among the 3,192 case reports of acute hepatitis B received by CDC for 2019, risk behavior and exposure data were missing for 1,183 (37.1%). Risk factors assessed under prior recommendations for HepB vaccination include potential criminal or stigmatizing behavior (e.g., injection-drug use, incarceration, or multiple sex partners), limiting the effectiveness of provider risk assessment (*3*,*12*,*13*). A universal vaccination recommendation eliminates the need for risk assessment before vaccination.

Racial and ethnic disparities exist among those who become infected with HBV. In 2005, acute hepatitis B incidence among non-Hispanic Black Americans was approximately twice that among several other racial and ethnic populations (*3*). In 2019, the rate of HBV infection among non-Hispanic Black adults was triple that of Asian or Pacific Islander adults and approximately twice that of Hispanic adults (*3*). Rates of hepatitis B among children and adolescents of all races and ethnicities converged to a lower rate after a universal vaccination strategy was implemented for this age group (*3*).

## Resource Use

An economic model was used to estimate the health improvements that are expected to result from universal adult HepB vaccination (*14*). One measure of cost-effectiveness, the incremental cost-effectiveness ratio (ICER), was calculated at $153,000 per quality-adjusted life-year (QALY) gained for all adults aged ≥19 years. A sub-analysis performed for adults aged 19–59 years yielded an ICER of $117,000 per QALY gained.^§^ Increased vaccination coverage resulting from the modeled vaccination intervention strategies resulted in better health outcomes; the average QALYs gained, life-years gained, number of acute HBV infections averted, and number of hepatitis B-related deaths averted all increased as vaccination coverage in the intervention strategy increased (*14*). Among the cohort aged ≥60 years, hepatitis B incidence is markedly lower (0.6 cases per 100,000 population in 2019); thus, the number of preventable HBV infections in that age group is lower than for those aged 19–59 years.

## Recommendations

HepB vaccination is recommended for adults aged 19–59 years and adults aged ≥60 years with risk factors for hepatitis B. Adults aged ≥60 years without known risk factors for hepatitis B may also receive HepB vaccines (Box). Infants and all other persons aged <19 years are already recommended to receive HepB vaccines (*2*).

BOX **Persons recommended to receive hepatitis B vaccination**

**All infants**

**Persons aged <19 years**

**Adults aged 19–59 years**

**Adults aged ≥60 years with risk factors for hepatitis B:**

- Persons at risk for infection by sexual exposure
  - Sex partners of persons testing positive for HBsAg
  - Sexually active persons who are not in a long-term, mutually monogamous relationship (e.g., persons with more than one sex partner during the previous 6 months)
  - Persons seeking evaluation or treatment for a sexually transmitted infection
  - Men who have sex with men
- Persons at risk for infection by percutaneous or mucosal exposure to blood
  - Persons with current or recent injection drug use
  - Household contacts of persons testing positive for HBsAg
  - Residents and staff members of facilities for persons with developmental disabilities
  - Health care and public safety personnel with reasonably anticipated risk for exposure to blood or blood-contaminated body fluids
  - Persons on maintenance dialysis, including in-center or home hemodialysis and peritoneal dialysis, and persons who are predialysis
  - Persons with diabetes at the discretion of the treating clinician
- Others
  - International travelers to countries with high or intermediate levels of endemic hepatitis B virus infection (HBsAg prevalence of ≥2%)
  - Persons with hepatitis C virus infection
  - Persons with chronic liver disease (including, but not limited to, persons with cirrhosis, fatty liver disease, alcoholic liver disease, autoimmune hepatitis, and an alanine aminotransferase or aspartate aminotransferase level greater than twice the upper limit of normal)
  - Persons with HIV infection
  - Persons who are incarcerated

**Adults aged ≥60 years without known risk factors for hepatitis B may receive hepatitis B vaccines**

**Abbreviation:** HBsAg = hepatitis B surface antigen.

## Clinical Guidance

ACIP recommends that adults aged 19–59 years and adults aged ≥60 years with risk factors for hepatitis B should receive HepB vaccines, and that adults aged ≥60 years without known risk factors for hepatitis B may receive HepB vaccines. In previous HepB vaccine recommendations, providers were advised to administer HepB vaccine to all patients who requested it. The new language for adults aged ≥60 years without known risk factors is intended to prompt all providers to offer HepB vaccination to patients in that cohort, rather than wait for a patient to request vaccination, thus shifting the responsibility of initiating the consideration of HepB vaccination from the patient to the provider.

Persons who have completed a HepB vaccination series at any point or who have a history of HBV infection should not receive additional HepB vaccination, although there is no evidence that receiving additional vaccine doses is harmful.^¶^ However, there are cases where revaccination might be indicated as specified in the 2018 ACIP recommendation (e.g., nonresponder infants born to persons testing positive for hepatitis B surface antigen [HBsAg], health care providers, and persons on hemodialysis) (*2*). Providers should only accept dated records as evidence of HepB vaccination. Vaccination of persons immune to HBV infection because of current or previous infection or HepB vaccination does not increase the risk for adverse events. However, in settings in which the patient population has a high rate of previous HBV infection,** prevaccination testing, which may be performed concomitantly with administration of the first dose of vaccine, might reduce costs by avoiding complete vaccination of persons who are already immune. Prevaccination testing consists of testing for HBsAg, antibody to HBsAg (anti-HBs), and antibody to hepatitis B core antigen (anti-HBc). The presence of HBsAg indicates current HBV infection. The presence of anti-HBs is generally interpreted as indicating immunity, either from HepB vaccination after a complete series or after recovery from HBV infection. The presence of total anti-HBc indicates previous or ongoing infection with HBV. Detailed interpretations of serologic markers for HBV infection are available (*2*). Lack of access to serologic testing should not be a barrier to vaccination of susceptible persons, especially in populations that are difficult to reach. Testing is not a requirement for vaccination, and in settings where testing is not feasible, vaccination of persons recommended to receive the vaccine should continue (*2*).

The safety and effectiveness of Heplisav-B and PreHevbrio have not been established in adults on hemodialysis (Table). Data are not available to assess the effects of Heplisav-B and PreHevbrio on the breastfed infant or on milk production and excretion. Data on Heplisav-B and PreHevbrio are currently insufficient to inform vaccine-associated risks in pregnancy (*8*,*15*). Thus, providers should vaccinate pregnant women needing HepB vaccination with Engerix-B, Recombivax HB, or Twinrix.

**TABLE:** Recommended doses and schedules of hepatitis B vaccine for adults aged ≥18 years and persons aged 11–19 years, by vaccine type and age group*

HepB vaccine*/Age group, yrs	Dose (*μ*g)	Volume (mL)	Schedule
**Recombivax HB**			
11–15	10	1	2 doses at 0 and 4–6 mos^†^
11–19	5	0.5	3 doses at 0, 1, and 6 mos^†^
≥20	10	1	
Adults on hemodialysis and other immunocompromised adults aged ≥20	40	1	
**Engerix-B**			
11–19	10	0.5	3 doses at 0, 1, and 6 mos
≥20	20	1	
Adults on hemodialysis and other immunocompromised adults aged ≥20	40	2	4 doses at 0, 1, 2, and 6 mos^§^
**Heplisav-B**			
≥18^¶^	20	0.5	2 doses at 0 and 1 mos
**Twinrix** (HepA-HepB combination vaccine)			
≥18	20	1	3 doses at 0, 1, and 6 mos (standard) or 4 doses at 0 d, 7 d, 21–30 d, and 12 mos (accelerated)
**PreHevbrio** (ACIP-recommended in 2022)			
≥18^¶^	10	1	3 doses at 0, 1, and 6 mos

**Abbreviations:** ACIP = Advisory Committee on Immunization Practices; HepA = hepatitis A; HepB = hepatitis B.

* If the HepB vaccination schedule is interrupted, the series does not need to be restarted. If a 3-dose series is interrupted after the first dose, the second dose should be administered as soon as possible; the second and third doses should be separated by an interval of ≥8 weeks. If only the third dose has been delayed, it should be administered as soon as possible. The final dose of a 3-dose series must be administered ≥8 weeks after the second dose and ≥16 weeks after the first dose; the minimum interval between the first and second doses is 4 weeks. Inadequate doses of hepatitis B vaccine or doses received after a shorter-than-recommended dosing interval should be readministered, using the correct dosage or schedule. Vaccine doses administered ≤4 days before the minimum interval or age are considered valid. Because of the unique accelerated schedule for Twinrix (https://www.fda.gov/media/119351/download), the 4-day guideline does not apply to the first 3 doses of this vaccine when administered on a 0-day, 7-day, 21–30-day, and 12-month schedule. PreHevbrio (https://www.fda.gov/media/154561/download) is a three-antigen HepB vaccine approved by the Food and Drug Administration in 2021 and recommended by ACIP in 2022.

^†^A 2-dose schedule of Recombivax HB adult formulation (10 *μ*g) (https://www.fda.gov/media/74274/download) is licensed for children and adolescents aged 11–15 years. When scheduled to receive the second dose, persons aged ≥16 years should be switched to a 3-dose series, with doses 2 and 3 consisting of the pediatric formulation administered on an appropriate schedule.

^§^ Engerix-B (https://www.fda.gov/media/119403/download) for adults on hemodialysis and is administered as a series of 4 doses (2 mL each) as a single 2-mL dose or as two 1-mL doses on a 0-, 1-, 2-, and 6-month schedule. Recombivax HB for adults on dialysis is a 3-dose series.

^¶^ The safety and effectiveness of Heplisav-B and PreHevbrio have not been established in adults on hemodialysis. Data are not available to assess the effects of Heplisav-B and PreHevbrio on breastfed infants or on maternal milk production and excretion. Data on Heplisav-B (https://www.fda.gov/media/108745/download) and PreHevbrio are currently insufficient to inform vaccine-associated risks in pregnancy. Thus, providers should vaccinate pregnant persons needing HepB vaccination with Engerix-B, Recombivax HB, or Twinrix.

**Summary**

**What is already known about this topic?**

Vaccination with hepatitis B (HepB) vaccines shows well-established safety and efficacy. However, because of risk factor−based approaches of previous vaccination recommendations, coverage among adults has been suboptimal.

**What is added by this report?**

In addition to groups for whom HepB vaccination is already recommended, the Advisory Committee on Immunization Practices recommends that all adults aged 19–59 years should receive HepB vaccines.

**What are the implications for public health practice?**

Universal adult HepB vaccination through age 59 years removes the need for risk factor screening and disclosure and could increase vaccination coverage and decrease hepatitis B cases.

## Footnotes

* Recommendations for routine use of vaccines in children, adolescents, and adults are developed by the ACIP. ACIP is chartered as a federal advisory committee to provide expert external advice and guidance to the CDC Director on use of vaccines and related agents for the control of vaccine-preventable diseases in the civilian U.S. population. Recommendations for routine use of vaccines in children and adolescents are harmonized to the greatest extent possible with recommendations made by the American Academy of Pediatrics, the American Academy of Family Physicians (AAFP), and the American College of Obstetricians and Gynecologists (ACOG). Recommendations for routine use of vaccines in adults are harmonized with the recommendations of AAFP, ACOG, and the American College of Physicians. ACIP recommendations approved by the CDC Director become agency guidelines on the date published in the *Morbidity and Mortality Weekly Report*. https://www.cdc.gov/vaccines/acip

† The ACIP Hepatitis Vaccines Work Group comprises professionals from academic medicine (family medicine, internal medicine, pediatrics, obstetrics, infectious disease, occupational health, and preventive medicine specialists), federal and state public health entities, and medical societies.

§
https://www.cdc.gov/vaccines/acip/meetings/downloads/slides-2021-11-2-3/02-HepWG-weng-508.pdf

¶
https://www.cdc.gov/vaccines/hcp/acip-recs/general-recs/index.html

** https://cdafound.org/polaris/ (Accessed November 19, 2021).
